# The Effectiveness of a Multimodal Brain Empowerment Program in Mild Cognitive Impairment: A Single-Blind, Quasi-Randomized Experimental Study

**DOI:** 10.3390/jcm12154895

**Published:** 2023-07-26

**Authors:** Wonjun Oh, Haeun Park, Mark Hallett, Joshua (Sung) H. You

**Affiliations:** 1 Sports Movement Artificial Robotics Technology (SMART) Institute, Department of Physical Therapy, Yonsei University, Wonju 26493, Republic of Korea; sdc01047@naver.com (W.O.); evepark16@yonsei.ac.kr (H.P.); 2 Human Motor Control Section, National Institute of Neurological Disorders and Stroke (NINDS), National Institutes of Health (NIH), Bethesda, MD 20814, USA; hallettm@ninds.nih.gov

**Keywords:** mild cognitive impairment, multimodal, EEG, robotic-assisted gait training, transcranial direct current stimulation, music therapy, computerized cognitive therapy, light therapy, core breathing exercises

## Abstract

The present study aimed to determine a multimodal brain empowerment (MBE) program to mitigate the modifiable risk factors in mild cognitive impairment (MCI), and its therapeutic effects are unknown. MBE encompassing (1) tDCS, light therapy, computerized cognitive therapy (TLC) and (2) robot-assisted gait training, music therapy, and core exercise (REM) interventions were randomly assigned to 20 healthy young adults and 20 older adults with MCI. The electroencephalography (EEG) power spectrum and topographic event-related synchronization (ERS) analysis were used to assess intervention-related changes in neural activity during the MBE program. Outcome: The EEG results demonstrated that both multimodal TLC and REM decreased delta waves and increased theta, alpha, and beta waves (*p* < 0.001). ERS showed increased neural activation in the frontal, temporal, and parietal lobes during TLC and REM. Such enhanced neural activity in the region of interest supports potential clinical benefits in empowering cognitive function in both young adults and older adults with MCI.

## 1. Introduction

A 2020 report by the Lancet Commission on Dementia Prevention, Intervention, and Care stated that the age-related incidence of dementia has decreased because of improvements in potentially modifiable risk factors, such as education, hypertension, hearing impairment, smoking, obesity, depression, physical inactivity, diabetes, and low social contact [1]. Growing evidence on dementia prevention, intervention, and care suggests that, among these modifiable risk factors, physical exercise with or without robotic systems, social participation empowerment, light, computerized cognitive training, robot-assisted gait training (RAGT), music, computerized cognitive therapy, transcranial direct current stimulation (tDCS), light, and core breathing exercises, either alone or in combination, can prevent the progression of dementia in patients with mild cognitive impairment (MCI) [2]. Furthermore, a recent clinical trial [3] showed promising results regarding the efficacy of multimodal therapy, including personalized cognitive stimulation, neurofeedback training, and brain coaching/counseling, for eating a Mediterranean diet and consuming omega-3 supplements. The combination of different modalities produces beneficial functional (cognitive) and EEG neurophysiological assessment changes (e.g., increased alpha and beta waves) in the fronto-temporal lobe, supporting the importance of multimodal approaches to reverse or regenerate neural substrates in MCI [4]. In fact, there is growing EEG neurophysiological evidence that a combination of different modalities (e.g., combined computerized physical and cognitive training) helps empower cognitive brain function in MCI, which is manifested by increased beta waves in the fronto-temporal brain area [5].

Based on collective and effective neuroscientific evidence, we developed a multimodal brain empowerment (MBE) program that encompasses systematic physical resistance exercise with RAGT, music therapy, computerized cognitive therapy, tDCS, light therapy, and core breathing exercises [6,7,8,9,10,11,12,13,14]. The multimodal brain enhancement approach utilizes different sensory systems to target brain areas affected by dementia and aims to improve brain function through sensory stimulation and related cognitive activities. However, the immediate efficacy of this approach and its underlying neural mechanisms remain to be determined [15]. Hence, the present study aimed to determine the immediate effectiveness of the multimodal brain empowerment approach on EEG patterns and to ascertain the underlying neural mechanisms between the individual modality and combination therapies (a combination of tDCS, light therapy, and computerized cognitive therapy (TLC), and robot-assisted gait training, music therapy, and core exercise (REM)) in both young healthy adults and older adults with MCI. The basic premise is that both combinations would have similar effects on EEG patterns in young, healthy adults and older adults with MCI or those at a high risk of MCI.

## 2. Materials and Methods

### 2.1. Design

This study used a two-group pre-test–post-test design. The participants participated in the MBE program at the Yonsei Good Wellness Center in Wonju, Republic of Korea, between January 2022 and June 2022. To assess the baseline and post-intervention effects on the participants, a baseline test was conducted without any intervention with the eyes closed, followed by the sequential application of six different interventions and two randomly assigned combinations. Subsequently, the post-test was administered individually for each intervention or combination.

### 2.2. Participants

Forty participants, twenty older adults with MCI (mean ± standard deviation: 79 ± 8.25 years; 10 women) and twenty healthy adults (mean ± standard deviation: 25.2 ± 3.19 years; 11 women), were recruited from residents of Wonju, Gangwon-do, and signed an informed consent form. The recruitment method involved introducing the experiment directly to the local community in Wonju City and posting recruitment notices on community bulletin boards to encourage the voluntary participation of eligible individuals. The experimental protocol was approved by the Yonsei Institutional Review Board (IRB) and Ethics Committee (approval no. 1041849-202202-BM-033-02). The inclusion criteria were as follows: (a) age > 65 years; (b) a score between 20 and 23 points on the Korean version of the Mini-Mental State Examination (MMSE); (c) self-reported subjective decline in cognitive function, which was also confirmed by a family member; and (d) age-matched cognitive deficits (less than at least 1.5 standard deviation) in one or more formal cognitive tests, including the CIST and the MMSE [3]. The inclusion criterion for young adults was an age range of 18–35 years [16]. The exclusion criteria included: (a) currently diagnosed dementia; (b) specific abnormalities in the brain, such as focal brain lesions, as detected by head MRI or CT; (c) a history of mental illness or substance abuse before the onset of dementia; (d) complications of other neurological diseases or illnesses; and (e) the use of antipsychotics, antidepressants, or anxiolytic drugs [17].

### 2.3. Intervention—MBE Program

**Figure 1:**
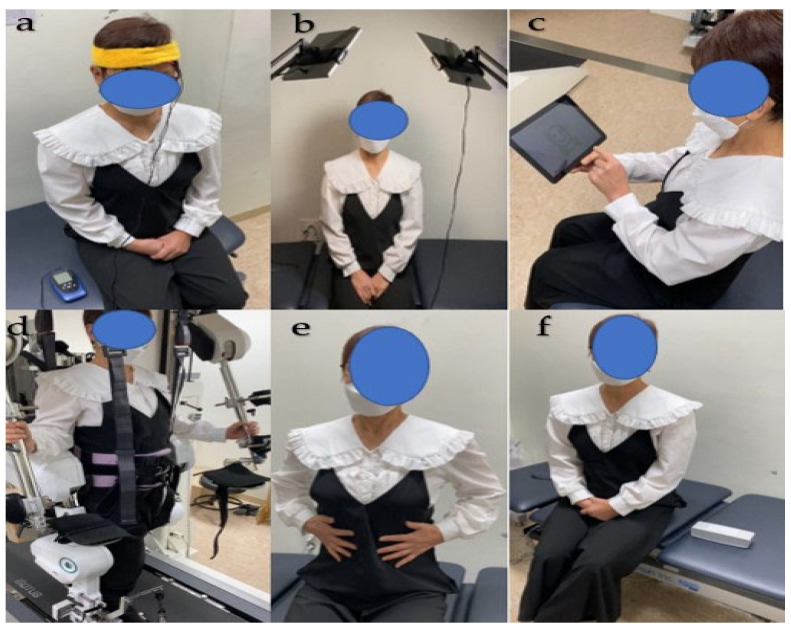
Six different interventional modalities. (**a**) tDCS; (**b**) light; (**c**) computerized cognitive therapy; (**d**) RAGT; (**e**) core breathing exercise; (**f**) music. Abbreviations: RAGT, robot-assisted gait training.

The MBE program included six different interventional modalities and two randomly administered combinations. The six different interventional modalities entailed (1) the application of 1 to 2 mA of transcranial direct current stimulation (tDCS), (2) exposure to 10,000 lx of light, (3) engagement in a medium-difficulty game targeting memory and attention through computerized cognitive therapy, (4) 20% body weight support during comfortable-speed robotic-assisted gait training (RAGT), and (5) during core breathing exercises, which involve respiration, the diaphragm descends to enhance intra-abdominal pressure (IAP). This in turn exerts outward pressure on the lower abdomen, thereby facilitating sufficient expansion. When exhaling, it is crucial to slightly retract the navel while maintaining this IAP. This ensures controlled exhalation at a consistent rate of 8–10 cycles per minute, promoting effective breathing techniques. Finally, (6) listening to relaxing music at a frequency of 40 Hz [2]. Except for RAGT, all other interventional modalities were performed in a comfortable sitting position. The RAGT, on the other hand, was conducted in a standing position (Figure 1).

**Figure 2:**
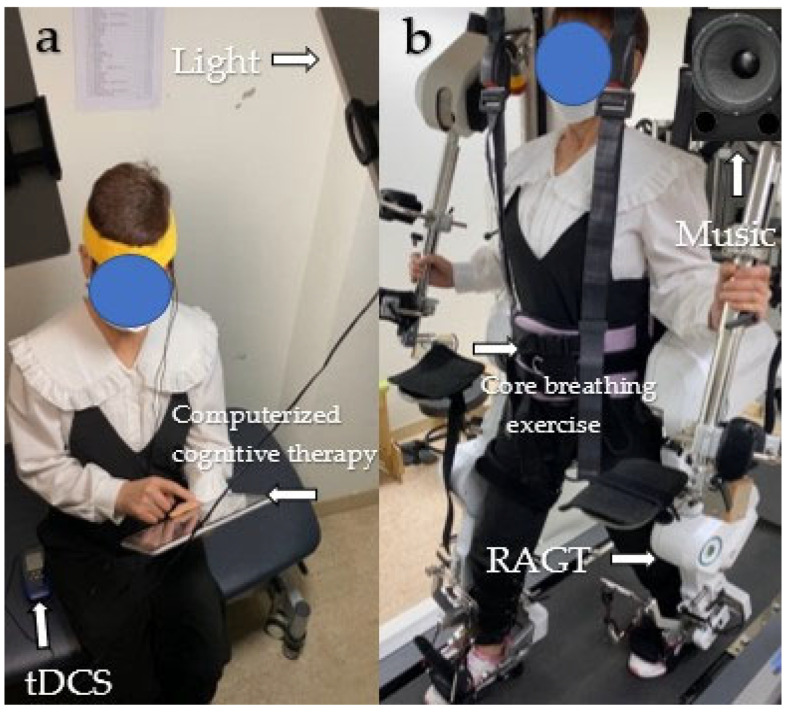
Two combinations of modalities. (**a**) TLC; (**b**) REM. Abbreviations: TLC, tDCS + light + computerized cognitive training; REM, RAGT + vore breathing exercise + music.

One combination involves simultaneous application of 1 to 2 mA of transcranial direct current stimulation (tDCS) and exposure to 10,000 lx of light while engaging in a medium-difficulty game targeting memory and attention through computerized cognitive therapy, all in a comfortable seated position. The other combination involves performing 20% body weight support during comfortable-speed robotic-assisted gait training (RAGT), core breathing exercises at a fixed rate of 8–10 cycles per minute, and listening to relaxing music at a frequency of 40 Hz while standing [2] (Figure 2).

The intervention dosage was standardized for six different interventional modalities and combinational interventional modalities, 60 s stimulation, followed by immediate EEG assessment (90 s) and a washout period (60 s), accounting for 210 s.

### 2.4. Clinical Outcome Measures

#### 2.4.1. EEG

A wireless 24-channel EEG measurement unit (g.Nautilus, g.tec Medical Engineering GmbH, Schiedlberg, Austria) with active electrodes on the cap was used to determine intervention-related synchronization (ERS) changes in the regions of interest (ROI).

During the MBE program, all participants underwent EEG measurements to evaluate changes in neural activity resulting from the intervention. The 32 active EEG electrodes were positioned on the scalp using a 10–20 system. The 24 EEG channels included Fp1, Fp2, Fz, F3, F4, F7, F8, FC1, FC2, FC6, T7, T8, Cz, C1, C2, C3, C4, C5, CP1, CP2, P2, P3, Pz, and POz, with a sampling rate of 500 Hz. The ground electrode was placed in the AFz region of the brain, and the reference electrode was placed in the right earlobe. An electrode gel was used for each electrode, and the resistance between the skin and the electrode was reduced to below 50 kΩ for each channel [18]. For the brain wave bands, frequencies, and functions, see Table 1.

#### 2.4.2. EEG Analysis

EEG processing was performed using the EEGLAB Toolbox [19]. To eliminate high-amplitude artifacts such as eye blinks and muscle bursts, we applied artifact subspace reconstruction (ASR) to the 24 channels that were bandpass filtered between 1 and 60 Hz. ASR implements principal component analysis on EEG data in sliding windows and detects channels that differ considerably from the baseline data containing minimal movement artifacts. An independent component analysis (ICA) was employed to separate the channels [20]. Finally, topographic mapping and ERD/ERS pattern analyses were performed.

### 2.5. Statistical Analysis

The results are presented as the mean and standard deviation. The sample size required for the study was determined using G* Power software (version 3.1; Düsseldorf, Germany). An effect size of 0.5 and a significance level (α) of 0.05 were utilized in the calculations. The results indicated that in order to achieve a statistical power of 0.8, a total of 40 subjects were required for the study [21]. All continuous variables were analyzed using the Kolmogorov–Smirnov test, and the assumption of normal distribution was satisfied. A paired *t*-test was used to analyze the EEG time–frequency power of each frequency band between the baseline condition and each respective condition. Demographic homogeneity was assessed using independent *t*-tests. SPSS for Windows (version 25.0, SPSS, Chicago, IL, USA) was used for statistical analyses. The alpha level was set at 0.05.

## 3. Results

Forty participants took part in the study, including twenty older adults with MCI (mean ± standard deviation: 79 ± 8.25 years; 10 women) and twenty young adults (mean ± standard deviation: 25.2 ± 3.19 years; 11 women). The average baseline MMSE scores for the older and young adults were 23 and 26, respectively (Table 2).

### 3.1. Delta Band

Young and older adults showed significant differences in time-frequency power between baseline and the eight interventions. In young adults, the mean delta power value was significantly lower in all interventions than in the baseline condition. In particular, tDCS, REM, and RAGT had the lowest mean delta frequencies compared to the other interventions. In older adults, the mean delta power value was significantly lower after TLC, computerized cognitive therapy, light, music, REM, tDCS, and RAGT. TLC, music, and computerized cognitive therapy showed the lowest mean value of delta compared with the other interventions (Table 3a,b).

### 3.2. Theta Band

In young adults, significant changes in time–frequency power were observed between baseline and all interventions, excluding CCT. In older adults, significant changes were observed with all interventions except for tDCS. The mean theta power value was significantly higher for all interventions in both young and older adults. In young adults, tDCS and TLC revealed the highest theta values compared with the other interventions. In older adults, theta power was the highest in REM, music, and core breathing exercises and showed the highest theta compared to other interventions (Table 3a,b).

### 3.3. Alpha Band

In young adults, significant changes in time–frequency power were observed between baseline and all interventions, except for core breathing exercises, CCT, and music. In older adults, significant changes were observed in all interventions, except for the core breathing exercises. In both young and older adults, the mean alpha power was significantly higher for all interventions. TLC and REM revealed the highest alpha values compared with the other interventions (Table 3a,b).

### 3.4. Beta Band

Both young and old adults revealed significant differences in time-frequency power between baseline and the eight interventions. In both young and older adults, the mean delta power was significantly higher for all interventions. In particular, TLC and REM revealed the highest beta values compared with the other interventions (Table 3a,b).

### 3.5. Topographic Maps

**Figure 3:**
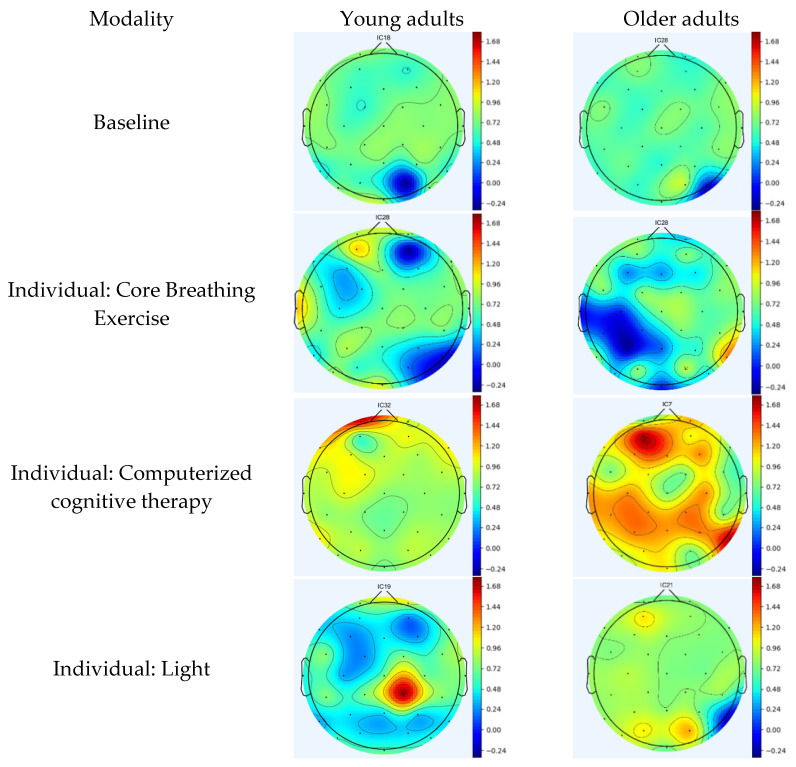

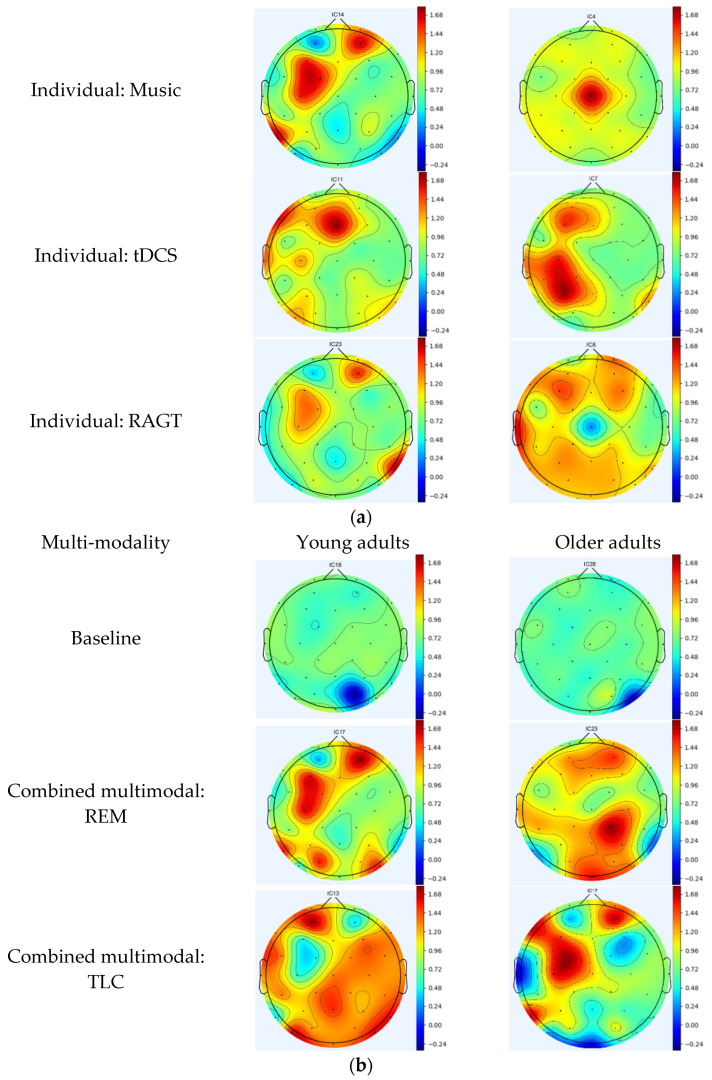
(**a**) Topographic maps following the application of individual modality. (**b**) Topographic maps following the application of multimodal brain empowerment program modality. Topographical distribution of selected EEG power spectra during standing and dual tasks. Dark and light blue and red colors indicate the power in each frequency band. The topographical distribution of the absolute spectral power (μV2/Hz) of alpha demonstrated a significant decrease in the central regions, and the theta and theta/beta ratio demonstrated a significant increase in all regions. Blue: ERD; red: ERS. ERD/ERS: event-related desynchronization/synchronization.

Using computerized cognitive therapy, young adults exhibit event-related synchronization (ERS) in the frontal region, which controls cognitive and memory functions [22]. In contrast, older adults demonstrate ERS activation in the frontal, temporal (primary functions include memory and understanding), and parietal (responsible for sensing and perception) regions [23,24]. During light therapy, young adults show ERS in the parietal region, whereas older adults show ERS in the occipital region (which serves as the center for processing visual stimuli) [25]. In music therapy, young adults demonstrate ERS in the frontal, parietal, and temporal regions, whereas older adults exhibit ERS in parietal areas. Young and older adults exhibit ERS in the frontal and parietal regions during REM. In TLC, young and older adults exhibit ERS in the frontal, temporal, and parietal regions of the brain. In tDCS, young and older adults exhibit ERS in both temporal and parietal regions. In the RAGT, young adults displayed ERS in the frontal, temporal, and parietal regions, whereas older adults showed ERS in the frontal, parietal, temporal, and occipital regions (Figure 3a,b).

## 4. Discussion

This study investigated the immediate effectiveness of an MBE program on qualitative (QL-EEG) and quantitative (QN_EEG) EEG patterns to ascertain the underlying neural mechanisms of six individual physical resistance exercises with RAGT, music therapy, computerized cognitive therapy, tDCS, light therapy, and core breathing exercise intervention modes, as well as two sets of combined intervention modes (TLC and REM), in healthy young adults and older adults with MCI. As hypothesized, the QN_EEG analysis suggested that both young and older adults with MCI showed more normalized EEG power spectrum patterns, as evidenced by a substantial decrease in delta values during the respective TLC and REM conditions, as well as more EEG power increases in theta, alpha, and beta values during all individual intervention modes compared to the baseline condition. The QL_EEG analysis indicated that both young and older adults with MCI showed more normalized ERS activation in the neural substrates representing the frontal, temporal, and parietal lobes during the individual intervention modes (RAGT, music therapy, computerized cognitive therapy, tDCS, light therapy, and core breathing exercises) and the TLC and REM combination modes. Most importantly, both the QL-EEG and QN_EEG results demonstrated promising effects of the individual MBE intervention mode, and more so with the combination mode. This was the first clinical trial to examine multimodal interventions for MCI, making it difficult to compare with the current literature.

The QN_EEG power spectrum analysis related to TLC indicated an increase in theta, alpha, and beta values in young adults and a decrease in delta values along with an increase in alpha and beta values in older adults. This finding is consistent with those of previous studies. Westwood et al. (2022) reported that a combination of tDCS and cognitive training resulted in a 0.81% increase in theta and a 2.35% increase in alpha brainwaves in 23 young participants, whereas beta brainwaves remained stable [26]. Andrade et al. (2022) found that multisite anodal transcranial direct current stimulation combined with cognitive stimulation induced changes in EEG spectral power at high (alpha and beta) and low (delta) frequencies in 36 older adults with Alzheimer’s disease [27]. The QL-EEG power spectrum analysis revealed that young and older adults primarily exhibited ERS in the frontal, temporal, and parietal regions. This result is consistent with those of previous studies. Dong et al. (2020) reported that a combination of tDCS and working memory training resulted in ERS in the frontal and parietal lobes in 34 young adults [28]. Münch et al. (2014) demonstrated that exposure to bright light resulted in ERS in the frontal lobes of 16 healthy adults [29]. A possible rationale for these results is that the combination of tDCS, light, and cognitive training may have synergistic effects on neural activity [30,31]. tDCS may enhance cortical excitability, light may modulate circadian rhythms and/or activate neural circuits involved in cognitive processing, and cognitive training may promote learning and memory consolidation. These factors may lead to the creation of a new cortical organization by increasing the activity of the frontotemporal and parietal lobes [32].

Analysis of the QN_EEG power spectrum in relation to REM showed a decrease in delta and an increase in alpha and beta values in young adults and an increase in theta, alpha, and beta values in older adults. This finding is consistent with previous evidence. Possti and colleagues (2020) found that dual-task walking decreased delta power by 45.83% and increased alpha power by 125% compared to single-task walking in a sample of 10 healthy adults [33]. Additionally, in 10 older adults, dual-task walking increased alpha power by 53.85% and beta power by 25% compared to single-task walking. The QL-EEG power spectrum analysis revealed that young and older adults exhibited ERS in the frontal and parietal regions. These data are consistent with those reported in previous studies. Breitling et al. (1987) reported that music activated brain mapping in the frontal lobes in 22 young adults [34]. Formaggio et al. (2017) observed ERS in the frontal and parietal lobes of 21 middle-aged adults during robot-assisted foot movements [35]. Patnaik et al. (2022) demonstrated that breathing exercises activated the frontal region using topographic maps of 14 young adults [36]. Ferrarelli et al. (2013) demonstrated that mindful breathing meditation led to ERS in the parietal regions of 29 middle-aged adults [37]. A possible rationale for these results is that the combination of RAGT, music, and breathing exercises may have a cooperative effect on neural activity [30]. Music and breathing exercises may enhance attention and relaxation, respectively, whereas RAGT may require attention and cognitive effort. These factors may increase the activity of the frontal and parietal lobes, thereby stimulating neural activity and contributing to increased cortical network activation [38].

Some limitations of this study should be considered for future studies. One limitation is that this preliminary clinical study examined the effects of individual and MBE intervention modes on neurophysiological phenomena using both QEEGs. However, further investigation of the structural and functional cortical changes in the involved neural substrates with advanced functional MRI imaging in a long-term cohort interventional study is warranted to validate the long-term effects of MBE. Another limitation is that this preliminary experiment only measured the immediate effects of brief interventions and had a brief washout period based on the participants’ tolerance and safety.

## 5. Conclusions

This clinical study demonstrated that both the young and old groups showed a decrease in delta waves during TLC and REM, along with an increase in theta, alpha, and beta waves. Topographic maps also revealed significant event-related synchronization (ERS) in the frontal, temporal, and parietal lobes of the brain during TLC and REM. These findings offer clinical evidence-based insights into the use of TLC and REM, which not only enhance cognitive function but also activate the frontal, temporal, and parietal lobes in both young adults and individuals with MCI.

## Figures and Tables

**Table 1:** The brain wave bands, frequency, and functions.

Brainwaves	Frequency (Hz)	Functions
Delta	2–4 Hz	Deep sleep, dreaming, and coma
Theta	4–8 Hz	Drowsy, meditation, and mental imagery
Alpha	8–12 Hz	Relaxed, calm, and lucid
Beta	12–30 Hz	Concentration, awake, alert, thinking, and mental activity

**Table 2:** Demographic characteristics (*N* = 40).

Characteristics	Older Adults with MCI (*n* = 20)	Young Adults (*n* = 20)	*p*-Value
Age (years)	79 ± 8.25	25.2 ± 3.19	0.006 *
Height (cm)	157.25 ± 8.45	169.65 ± 9.37	0.717
Weight (kg)	59.4 ± 7.3	67.7 ± 13.83	0.002 *
MMSE	21	26	0.000 *
Gender (M/F)	10/10	9/11	0.664

The values are presented as the mean ± standard deviation. Abbreviations: MMSE, Mini-Mental State Examination; * *p*-value obtained by an independent *t*-test.

**Table 3:** (**a**) Time–frequency power spectrum of EEG—young adults. (**b**) Time–frequency power spectrum of EEG—older adults.

(a)			
	Young Adults		*p*-Value
Delta, 2–4	Baseline	Conditions	
Core breathing exercise	2.64 ± 0.32	2.36 ± 0.20	0.000 **
Computerized cognitive therapy	2.64 ± 0.32	1.46 ± 0.70	0.000 **
Light	2.64 ± 0.32	1.50 ± 0.21	0.000 **
Music	2.64 ± 0.32	1.79 ± 0.64	0.000 **
REM	2.64 ± 0.32	1.22 ± 0.47	0.000 **
tDCS	2.64 ± 0.32	1.22 ± 0.14	0.000 **
TLC	2.64 ± 0.32	1.45 ± 0.33	0.000 **
RAGT	2.64 ± 0.32	1.17 ± 0.33	0.000 **
Theta, 4–8	Baseline	Conditions	
Core breathing exercise	0.41 ± 0.37	1.07 ± 0.59	0.000 **
Computerized cognitive therapy	0.41 ± 0.37	0.48 ± 0.14	0.876
Light	0.41 ± 0.37	0.81 ± 0.48	0.000 **
Music	0.41 ± 0.37	0.91 ± 0.24	0.000 **
REM	0.41 ± 0.37	1.10 ± 0.31	0.000 **
tDCS	0.41 ± 0.37	1.29 ± 0.18	0.000 **
TLC	0.41 ± 0.37	1.28 ± 0.27	0.000 **
RAGT	0.41 ± 0.37	0.74 ± 0.36	0.000 **
Alpha, 8–12	Baseline	Conditions	
Core breathing exercise	−0.51 ± 0.41	−0.45 ± 0.51	0.376
Computerized cognitive therapy	−0.51 ± 0.41	−0.37 ± 0.25	0.060
Light	−0.51 ± 0.41	−0.13 ± 0.22	0.000 **
Music	−0.51 ± 0.41	−0.38 ± 0.40	0.053
REM	−0.51 ± 0.41	0.52 ± 0.26	0.000 **
tDCS	−0.51 ± 0.41	0.16 ± 0.37	0.000 **
TLC	−0.51 ± 0.41	0.77 ± 0.21	0.000 **
RAGT	−0.51 ± 0.41	−0.28 ± 0.22	0.001 **
Beta, 12–30	Baseline	Conditions	
Core breathing exercise	−0.56 ± 0.30	−0.23 ± 0.28	0.000 **
Computerized cognitive therapy	−0.56 ± 0.30	−0.11 ± 0.31	0.000 **
Light	−0.56 ± 0.30	−0.17 ± 0.21	0.000 **
Music	−0.56 ± 0.30	−0.03 ± 0.26	0.000 **
REM	−0.56 ± 0.30	0.09 ± 0.20	0.000 **
tDCS	−0.56 ± 0.30	−0.012 ± 0.27	0.000 **
TLC	−0.56 ± 0.30	0.12 ± 0.18	0.000 **
RAGT	−0.56 ± 0.30	−0.2 ± 0.23	0.000 **
**(b)**			
	**Older Adults**		***p*-Value**
Delta, 2–4	Baseline	Conditions	
Core breathing exercise	2.62 ± 0.27	2.81 ± 0.31	0.000 **
Computerized cognitive therapy	2.62 ± 0.27	1.05 ± 0.29	0.000 **
Light	2.62 ± 0.27	1.86 ± 0.11	0.000 **
Music	2.62 ± 0.27	1.06 ± 0.53	0.000 **
REM	2.62 ± 0.27	1.55 ± 0.44	0.000 **
tDCS	2.62 ± 0.27	1.47 ± 0.23	0.000 **
TLC	2.62 ± 0.27	1.13 ± 0.41	0.000 **
RAGT	2.62 ± 0.27	2.22 ± 0.72	0.000 **
Theta, 4–8	Baseline	Conditions	
Core breathing exercise	0.39 ± 0.45	1.14 ± 0.13	0.000 **
Computerized cognitive therapy	0.39 ± 0.45	1.04 ± 0.17	0.000 **
Light	0.39 ± 0.45	0.87 ± 0.36	0.000 **
Music	0.39 ± 0.45	1.15 ± 0.61	0.000 **
REM	0.39 ± 0.45	1.43 ± 0.37	0.000 **
tDCS	0.39 ± 0.45	0.55 ± 0.52	0.326
TLC	0.39 ± 0.45	0.73 ± 0.54	0.000 **
RAGT	0.39 ± 0.45	0.56 ± 0.49	0.031 *
Alpha, 8–12	Baseline	Conditions	
Core breathing exercise	−0.67 ± 0.19	−0.60 ± 0.31	0.268
Computerized cognitive therapy	−0.67 ± 0.19	0.16 ± 0.12	0.000 **
Light	−0.67 ± 0.19	−0.46 ± 0.38	0.016 *
Music	−0.67 ± 0.19	−0.12 ± 0.62	0.000 **
REM	−0.67 ± 0.19	0.32 ± 0.51	0.000 **
tDCS	−0.67 ± 0.19	−0.43 ± 0.55	0.003 **
TLC	−0.67 ± 0.19	0.23 ± 0.54	0.000 **
RAGT	−0.67 ± 0.19	−0.23 ± 0.28	0.001 **
Beta, 12–30	Baseline	Conditions	
Core breathing exercise	−0.41 ± 0.21	−0.30 ± 0.25	0.007 **
Computerized cognitive therapy	−0.41 ± 0.21	0.04 ± 0.32	0.000 **
Light	−0.41 ± 0.21	−0.33 ± 0.14	0.022 *
Music	−0.41 ± 0.21	−0.23 ± 0.27	0.000 **
REM	−0.41 ± 0.21	0.08 ± 0.33	0.000 **
tDCS	−0.41 ± 0.21	−0.26 ± 0.24	0.003 **
TLC	−0.41 ± 0.21	0.09 ± 0.27	0.000 **
RAGT	−0.41 ± 0.21	−0.28 ± 0.41	0.001 **

Data are presented as mean the ± standard deviation. Abbreviations: tDCS, transcranial direct current stimulation; RAGT, robot-assisted gait training; TLC, tDCS + light + computerized cognitive training; REM, RAGT + core breathing exercise + music; *p*-value obtained using a paired *t*-test; * *p* < 0.05. ** *p* < 0.01.

## Data Availability

The data presented in this study are available from the corresponding author upon request.
